# The prophylactic effects of BIFICO on the antibiotic-induced gut dysbiosis and gut microbiota

**DOI:** 10.1186/s13099-020-00379-0

**Published:** 2020-09-09

**Authors:** Jiannong Wu, Tieer Gan, Ying Zhang, Guolian Xia, Shu Deng, Xin Lv, Bingxin Zhang, Bin Lv

**Affiliations:** 1 Department of Critical Care, The First Affiliated Hospital of Zhejiang Chinese Medical University, Hangzhou, China; 2 Department of Infection Management, The First Affiliated Hospital of Zhejiang Chinese Medical University, Hangzhou, China; 3 Center of Clinical Evaluation, The First Affiliated Hospital of Zhejiang Chinese Medical University, Hangzhou, China; 4 Department of Hematology, The First Affiliated Hospital of Zhejiang Chinese Medical University, Hangzhou, China; 5 Department of Pneumology, The First Affiliated Hospital of Zhejiang Chinese Medical University, Hangzhou, China; 6 Department of Pneumology, Fuyang District People’s Hospital of Hangzhou, Hangzhou, China; 7 Department of Gastroenterology, The First Affiliated Hospital of Zhejiang Chinese Medical University, 54 Youdian RoadZhejiang Province, Hangzhou, 310009 China

**Keywords:** AIGD, BIFICO, 16S rDNA sequencing, Gut microbiota, Antibiotics

## Abstract

**Background:**

The aim of this study is to evaluate the prophylactic effects of probiotic mixture BIFICO on antibiotic-induced gut dysbiosis (AIGD) and the influence on the change of the gut microbiota.

**Methods:**

We conducted a prospective, randomized, controlled study and divided 196 patients who required intravenous beta-lactam antibiotics into three groups: a control group (no probiotics), a regular group (840 mg of BIFICO), and a double-dosage group (1680 mg of BIFICO). The symptoms of antibiotic-related diarrhea, bloating and abdominal pain and the incidence of AIGD were evaluated 7 days and 8–14 days after antibiotic use, with 10 patients in each group. 16S rDNA sequencing was performed to detect changes of the gut microbiota.

**Results:**

Within 7 days of the initiation of antibiotic treatment, the incidences of AIGD in the control group, regular group (840 mg of BIFICO), and double-dosage group (1680 mg of BIFICO) were 21.88%, 14.93%, and 6.15% respectively. On days of 8–14th, the incidences of AIGD in the control group, regular group, and double-dosage group were 25%, 14.93%, and 4.62%, respectively. The incidence of AIGD in the double-dosage group within 7 days and 14 days were both significantly lower than that in relevant control group (P < 0.05). On day 14, the incidence of AIGD in the double-dosage group was lower than that in the regular group (P < 0.05). The number of operational taxonomic units (OTUs) in the control group after antibiotic treatment was significantly reduced compared to that prior to treatment, while those of the regular and double-dosage groups were stable. The species abundance, especially *Parabacteroides, Phascolarctobacterium* and *Roseburia*, of the double-dosage group was greater than that of the regular group and the control group.

**Conclusions:**

BIFICO may reduce the occurrence of AIGD in a dose-dependent manner and can stabilize the gut microbiota balance.

## Background

Antibiotics kill or inhibit pathogenic bacteria to treat countless patients [1]. However, antibiotics can also kill bacteria that normally colonize the human body, such as in the gut, and different types of antibiotics produce different effects on the composition and functioning of the gut microbiota [2]. Microbiota means the entire population of microorganisms that colonizes a particular location, and includes bacteria and other microbes such as fungi, archaea, viruses, and protozoan [3]. Gut microbiota maintains the gut in the normal individual and human health as a whole [4]. The disorder of the gut microbiota causes gut dysbiosis and results in a loss of taxonomic and functional diversity as well as reduced colonization by pathogens [5]. Antibiotic-mediated gut dysbiosis are related to diverse pathological conditions, including obesity, type 2 diabetes, inflammatory bowel disease, anxiety, autism, allergies, and autoimmune diseases [6], hippocampal neuroglial reorganization and depression [7], alzheimer's disease [8]. This increases medical costs and the length of hospitalization as well as severely threatens the lives of patients.

Probiotics are live microorganisms that are beneficial to the body and have been proven to be effective in preventing antibiotic-associated diarrhoea [9]. A recent study found that an oral probiotic combination of *Bifidobacterium* and *Lactobacillus* can alter the gut microbiota during antibiotic treatment for *Clostridium difficile* infection [10]. However, most of the related studies are clinical observations and analyses [11, 12], and there have been very few studies focusing on how probiotics can affect or regulate the gut microbiota.

BIFICO is a type of probiotic mixture containing *Bifidobacterium*, *Lactobacillus acidophilus*, and *Enterococcus faecalis*. Our study aimed to determine the effects of BIFICO on the prevention of antibiotic-induced gut dysbiosis. In addition, we analysed the effects of BIFICO on the gut microbiota balance with 16S rDNA sequencing.

## Methods

### Trial design and oversight

This trial was an open-label, prospective, randomized trial conducted in our hospital from January 2016 through December 2017. The Committee on the Ethics of Human Research of The First Affiliated Hospital of Zhejiang Chinese Medical University approved the study protocol (2016-ZX-014–02), which is available at the Chinese Clinical Trial Registry (ChiCTR1800015874). All methods were carried out in accordance with relevant guidelines and regulations. The written informed consent was obtained from all patients or their legal guardian about the trial, and they were informed that they could decline to participate at any time.

The trial was overseen by a steering committee that was presented with information regarding the rate of inclusion of new patients by the investigators by meeting every 3 months during the study period. An investigator at each inpatient ward was responsible for enrolling patients, ensuring adherence to the protocol, and completing the case-report form. Two residents vouched for the accuracy and the completeness of the reported data as well as for the adherence of the trial to the protocol. All analyses were performed by a clinical epidemiologist in accordance with the International Conference on Harmonisation Good Clinical Practice guidelines.

### Patients

Patients with bacterial infectious diseases who were hospitalized at the First Affiliated Hospital of Zhejiang Chinese Medical University were enrolled in this study if they fulfilled the following inclusion criteria: (1) the patients had a respiratory tract infection, urinary tract infection, or bloodstream infection; (2) the patients were expected to undergo intravenous administration of beta-lactam antibiotics for ≥ 7 days; (3) the patients were aged ≥ 18 years, with no gender restriction; and (4) the patients signed the informed consent forms, indicating their consent to participate in this study and their willingness to cooperate with the requirements and follow-up visits of this study.

The exclusion criteria were as follows: (1) patients who experienced diarrhoea within 2 weeks before intervention with BIFICO, including diarrhoea due to any cause (no stool formation ≥ 3 times/24 h); (2) patients with refractory constipation or patients who were currently on laxatives; (3) patients who underwent radiotherapy and chemotherapy within the last month; (4) patients who eat yogurt, drink probiotic beverages, etc., in their daily life; and (5) patients who are allergic to probiotics.

### Randomization

The patients who fulfilled the enrolment criteria were randomized into three groups: the control group, the regular group, and the double-dosage group. Each group contained 75 patients, corresponding to the sample size estimation. A random number was generated using a random number table. According to the hospitalization sequence, each subject was given a random number. This number was divided by three, and the patients were divided into groups that corresponded to the remainder of this division. When the number of patients in each group was not equal, a randomized adjustment was carried out again so that each group had the same number of patients.

### Interventions

The intervention was BIFICO (Bifico Pharmaceuticals, Sine, Shanghai, China). Each capsule is 210 mg, which contains approximately 1.0 × 10^9^ cfu/g of viable lyophilized bifidobacteria (*Bifidobacterium longum*), 1.0 × 10^9^ cfu/g lactobacilli (*Lactobacillus acidophilus*), and 1.0 × 10^9^ cfu/g *Enterococcus faecalis*. The control group didn’t receive any probiotics. The regular group was orally administered BIFICO twice a day for 14 days with 840 mg dose (the regular dose of 4 capsules in clinic) each time; and the double-dosage group was orally administered BIFICO twice a day for 14 days with a 1680 mg dose (double of regular dose commonly used for gut microbiota disorder in clinic) each time, according to the specifications.

### Outcomes

The duration of the follow-up for each patient was 14 days from the start of the intervention. The outcomes that were assessed were the incidence of AIGD and the change in the gut microbiota.

The diagnostic criteria for the incidence of AIGD were as follows [13]: (1) short-term or currently on antibiotics; (2) clinical presentation of diarrhoea, abdominal distension, abdominal pain, abdominal discomfort, or other symptoms of gut dysbiosis; and (3) laboratory results for gut dysbiosis. The laboratory results for gut dysbiosis included the following: (a) ratio of cocci/bacillus greater than 1/3 on stool microscopy examination, which is one of the diagnostic criteria for antibiotic-related intestinal flora disorders [14]; and (b) significantly elevated non-normal bacterial counts by faecal bacterial smear, culture, or microbiota gene testing, or dominance by non-normal bacteria. The aforementioned items (1) and (2) can be used as a basis for clinical diagnosis, as these are necessary conditions for gut dysbiosis. Gut dysbiosis can be diagnosed if any item in the laboratory testing category is detected. Patients with diarrhoea caused by acute episodes of chronic gastritis or acute gastrointestinal infection were excluded. According to the Gastrointestinal Symptom Rating Scale score [15], patients with an abdominal pain score ≥ 2 were considered to have abdominal pain, while patients with an abdominal distension score ≥ 2 were considered to have abdominal distension.

Ten patients were randomly selected from each group, and stool samples were collected before the intervention and on day 7 and day 14 after the intervention. Sterile containers (30 mL) were used for collecting the stool samples. The quantity of each stool sample was at least 10 g. Each stool sample was divided into three small test tubes (2 mL) and stored at − 80 °C for no more than one year. One tube was used for 16S rDNA sequencing, while the other two tubes were used for validation.

16S rDNA sequencing was performed to detect changes in the gut microbiota. Testing, including total DNA isolation, 16S amplicon sequencing, the construction of the gene catalogue, diversity analysis, taxonomic assignment and functional characterization, the determination of the bacterial DNA quality, and 16S ribosomal RNA amplicon sequencing, was carried out by BGI Tech Solutions Co., Ltd.

### Statistical analysis

For statistical analysis, SPSS 22.0 software was used. Normally distributed quantitative data are expressed as the mean ± standard deviation, while quantitative data that had a skewed distribution are expressed using the median and quartiles. Categorical variables are expressed as the number of cases/total number (%). Analysis of variance (ANOVA) was used for the comparison of markers among the three groups. Repeated-measures ANOVA was employed to determine the alpha diversity parameters and community richness at three different timepoints. The χ^2^ test was used for qualitative data. The significance level was set at α = 0.05.

## Results

### Patients

**Fig. 1:**
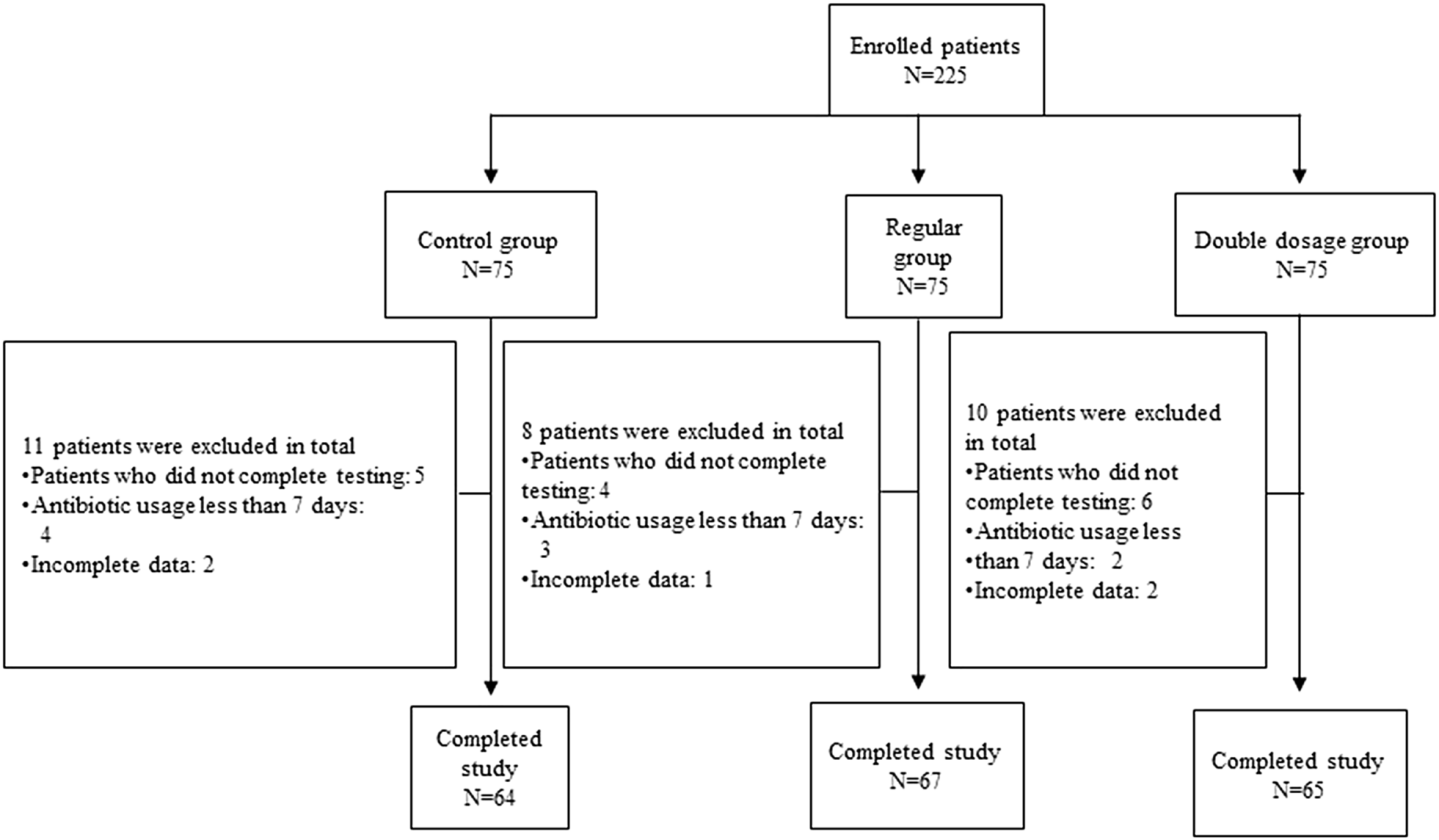
Flowchart of the study

Based on the inclusion and exclusion criteria, a total of 225 patients (75 patients per group) were enrolled. Twenty-nine patients dropped out of this study due to an insufficient antibiotic treatment course, uncompleted tests, or incomplete data. There were 196 patients with complete data: 64 patients in the control group, 67 patients in the regular group, and 65 patients in the double-dosage group (Fig. 1). The characteristics of the patients were well balanced among the three study groups, that there were no significant differences in ages, levels of inflammation markers (WBC, CRP, PCT), length of hospitalization, duration of antibiotic usage, types of antibiotic (carbapenem and non-carbapenem) (Table 1).

**Table 1:** Comparison of the general status of the patients from the three groups before the intervention

Parameter	Control group (n = 64)	Regular group (n = 67)	Double-dosage group (n = 65)	P-value
Age (years) (x ± s)	62.83 ± 16.97	66.99 ± 12.93	66.89 ± 13.92	0.187
Gender (Male) (n, %)	37 (57.81%)	35 (52.24%)	44 (67.69%)	0.189
Infection site (n) Respiratory system Non-respiratory system Inflammatory marker (x ± s)	28 36	31 36	24 41	0.533
WBC (10^9^ /L)	8.15 ± 3.29	8.40 ± 3.85	8.18 ± 3.69	0.911
CRP (mg/L)	54.72 ± 43.20	60.95 ± 41.32	59.09 ± 40.31	0.681
PCT (ng/mL)	0.20 ± 0.16	0.24 ± 0.17	0.23 ± 0.20	0.397
Length of hospitalization (days) M (P25, P75)	17.5 (12, 29.75)	19 (14, 33)	25 (15, 36)	0.081
Duration of antibiotic usage (days) (x ± s)	12.22 ± 4.84	11.21 ± 4.37	11.60 ± 4.09	0.426
Type of antibiotic (n) Carbapenem Non-carbapenem	50 14	47 20	49 16	0.566

*WBC* white blood cell count, *CRP* C-reactive protein, *PCT* procalcitonin

### Incidence of gut dysbiosis

The incidence of AIGD within 7 days and on days 8–14 in the control group was 21.88% and 25%, respectively. The incidence of AIGD was reduced in the double-dosage group (6.2%) at an early stage (within 7 days) compared to that of the control group (P < 0.05). In addition, the incidence of AIGD was also reduced in the regular group (14.93%) and double-dosage group (4.62%) on days 8–14 compared to that of the control group (P < 0.05) (Table 2).

**Table 2:** Comparison of the incidence of gut dysbiosis in the patients from the three groups

	Control group (n = 64)	Regular group (n = 67)	Double-dosage group (n = 65)	P-value
Within 7 days				
Incidence of gut dysbiosis (%)	21.88	14.93	6.1^5^**	0.038*
Days 8–14				
Incidence of gut dysbiosis (%)	25.00	14.93	4.62**	0.005**

* P < 0.05, **P < 0.01 compared to Control group

### Changes in the gut microbiota operational taxonomic units (OTUs) and diversity after BIFICO administration

**Fig. 2:**
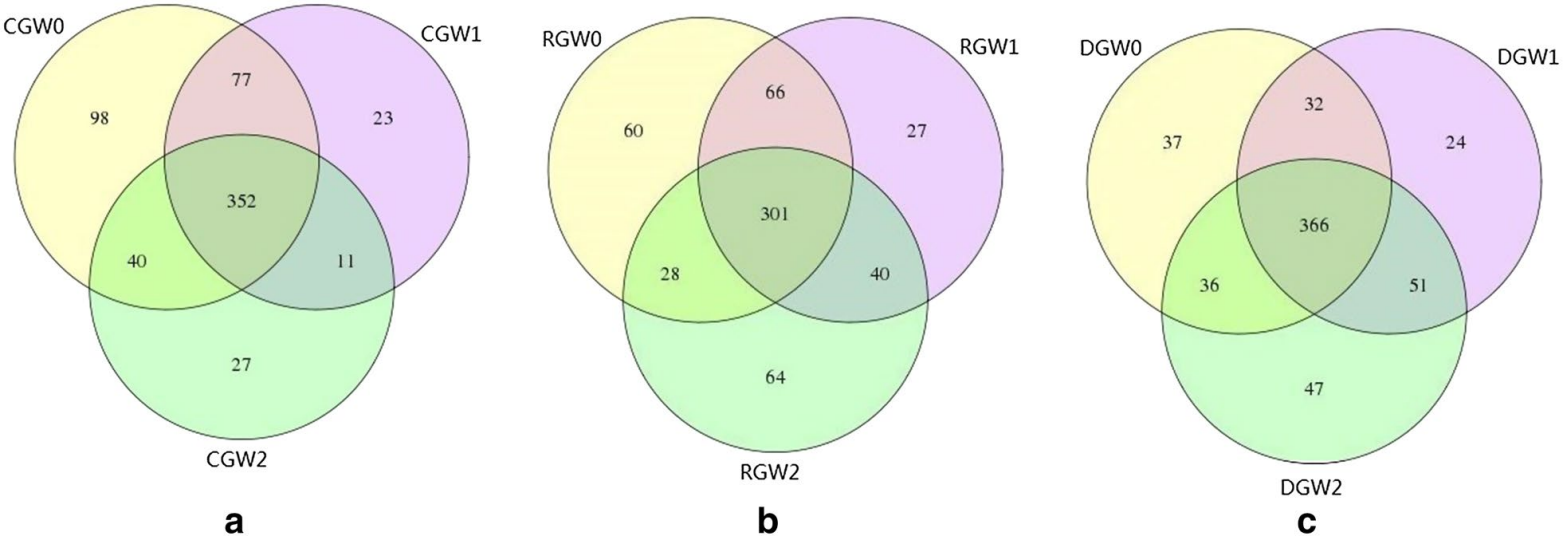
Venn diagram of OTU changes before and after intervention with BIFICO in the three groups. *CG* control group, *RG* regular group, *DG* double-dosage group, *W0* before intervention, *W1* day 7 after intervention, *W2* day 14 after intervention. The different colours in the figure represent different samples or different groups. The numbers in the overlapping sections between the different coloured circles represent the number of OTUs that were common to two samples or to two groups

**Fig. 3:**
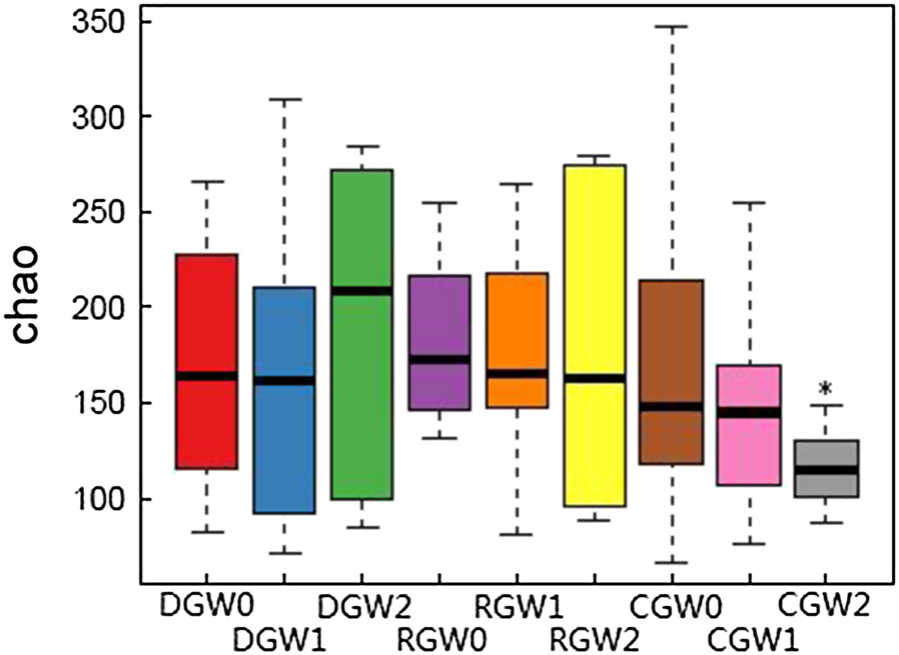
Chao index comparison between the three groups before the intervention, on day 7, and on day 14. Note: CG, control group; RG, regular group; DG, double-dosage group; W0, before intervention; W1, day 7 after intervention; W2, day 14 after intervention; *comparison between CGW2 and CGW0, P = 0.044

**Fig. 4:**
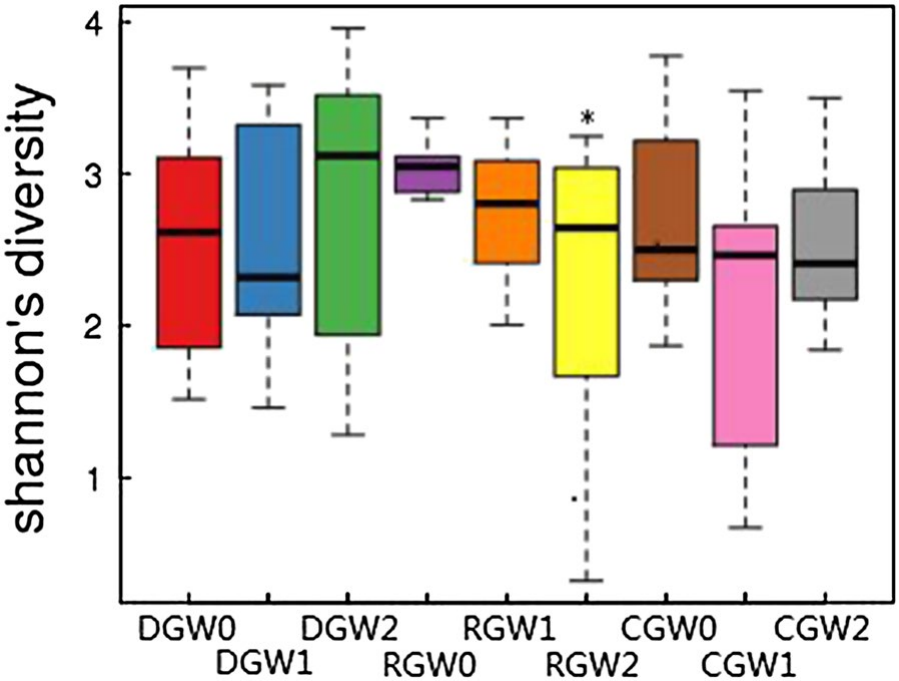
Box plot of the Shannon index before and after intervention with BIFICO in the three groups. Note: CG, control group; RG, regular group; DG, double-dosage group; W0, before intervention; W1, day 7 after intervention; W2, day 14 after intervention; *comparison between CGW2 and CGW0, P = 0.044

Venn diagram of OTU changes before and after intervention with BIFICO in the three groups before the intervention, on day 7, and on day 14 was determined (Fig. 2). A total of 7,954,570 sequences were obtained from 90 samples in this study, with an average of 88,384 sequences per sample. The mean length of each sequence was 252 bp. With 87% similarity, a total of 753 OTUs were identified from the 90 samples. The number of OTUs of the control group was significantly lower than that of the other groups, and this was mainly due to a reduced gut microbiota richness, compared by Chao index comparison among the three groups before the intervention, on day 7, and on day 14 were analysed (Fig. 3). Shannon index comparison among the three groups before the intervention, on day 7, and on day 14 were analysed, and there were no significant effects on the gut microecological structure and diversity (Fig. 4). Changes in the gut microecological abundance and the number of OTUs were not significant when different doses of BIFICO were given to the regular and double-dosage groups (Table 3).

**Table 3:** Chao index comparison between the three groups before the intervention, on day 7, and on day 14

	Before intervention	Day 7	Day 14	Intra-group differences	Before intervention vs. day 14 Paired t-test
Control group (n = 64)	181.09 ± 97.857	148.34 ± 86.414	131.01 ± 51.517	0.044	P = 0.089 P = 0.270 P = 0.046
Regular group (n = 67)	187.45 ± 53.514	177.73 ± 61.194	168.587 ± 81.087	0.453	
Double-dosage group (n = 65)	183.60 ± 69.870	177.77 ± 94.566	198.628 ± 93.137	0.476	
Intra-group differences	0.982	0.655	0.166		

### Species distribution among the three groups

**Fig. 5:**
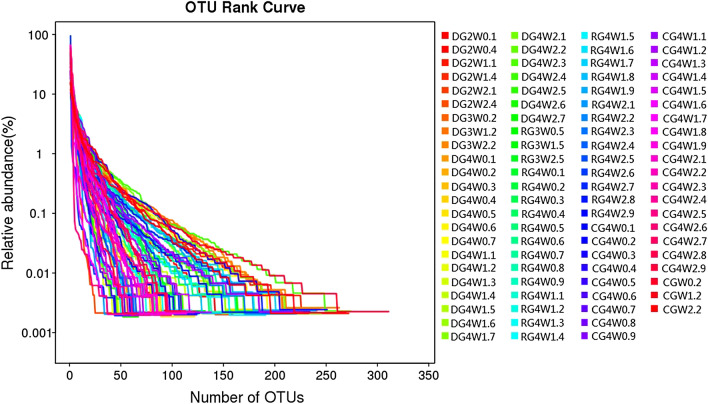
Rank curves of the OTUs in the three groups. *CG* control group, *RG* regular group, *DG* double-dosage group

As shown by the x-coordinates of the OTU rank curve (Fig. 5), the species abundance of the double-dosage group was greater than that of the regular group, and the species abundance of the regular group was greater than that of the control group. However, we were unable to find differences in the abundance between the subgroups in the various groups. In addition, the curves of the three groups are not flat. Therefore, the species distribution of the three groups (control, regular, and double-dosage groups) is not uniform.

### Bacterial flora analysis at various taxonomic levels in the three groups

**Fig. 6:**
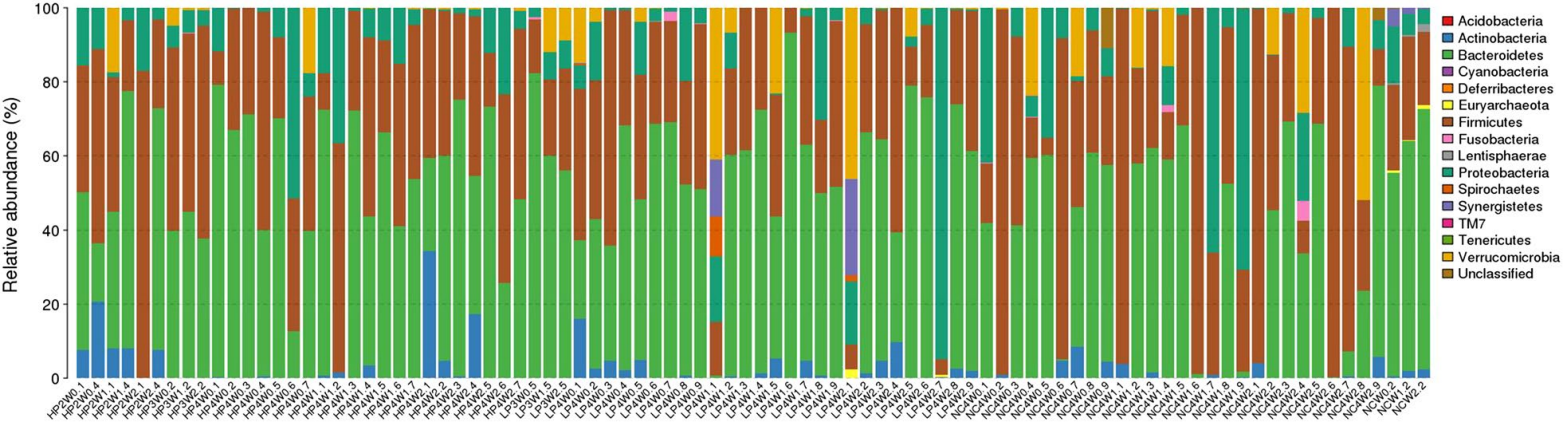
Histogram of the phylum profile

**Fig. 7:**
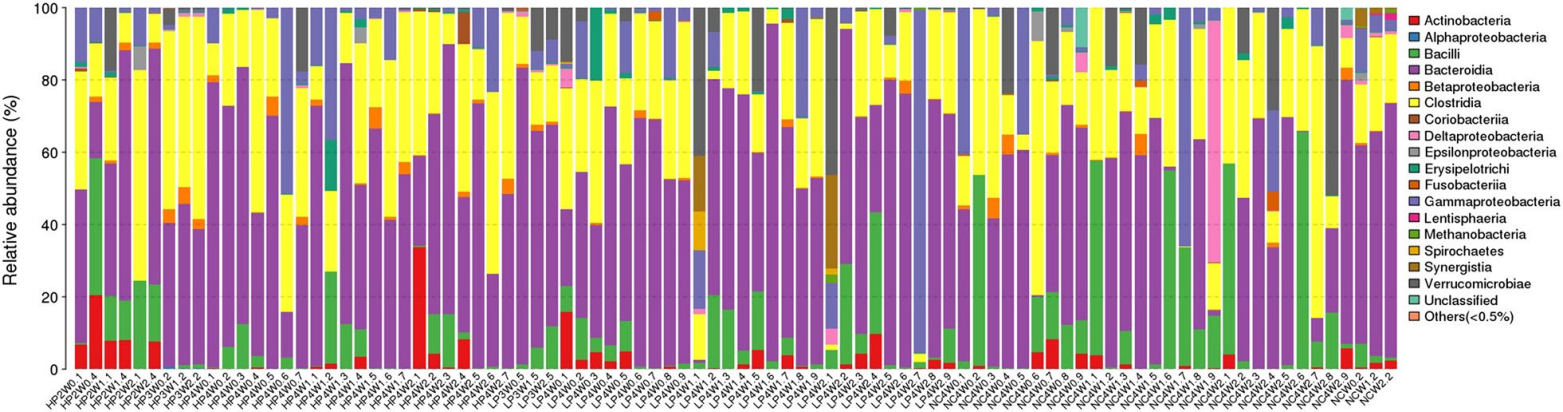
Histogram of the class profile

**Fig. 8:**
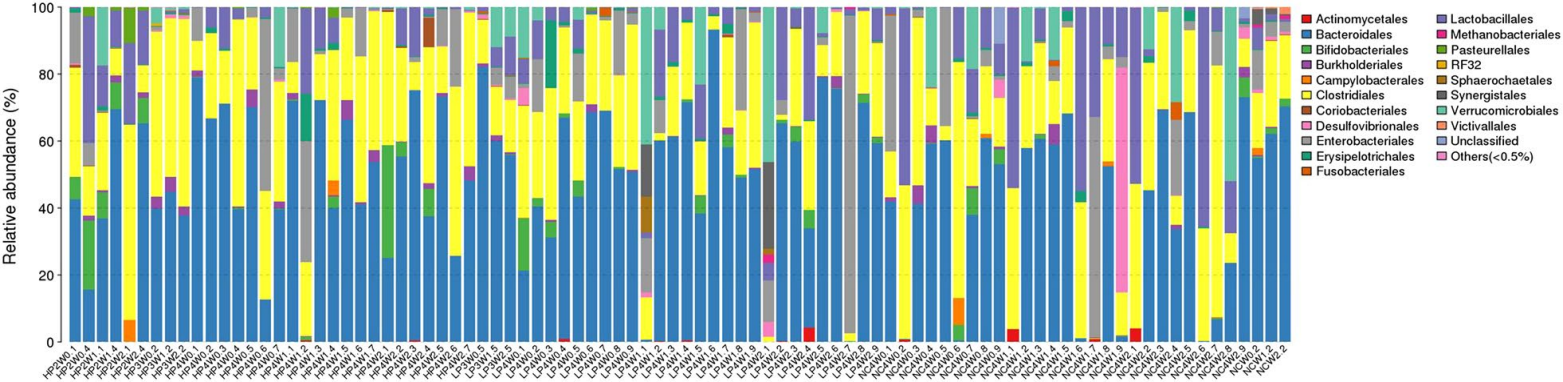
Histogram of the order profile

**Fig. 9:**
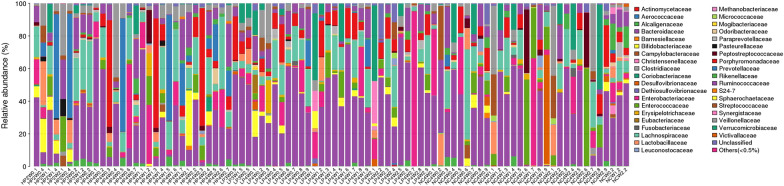
Histogram of the family profile

**Fig. 10:**
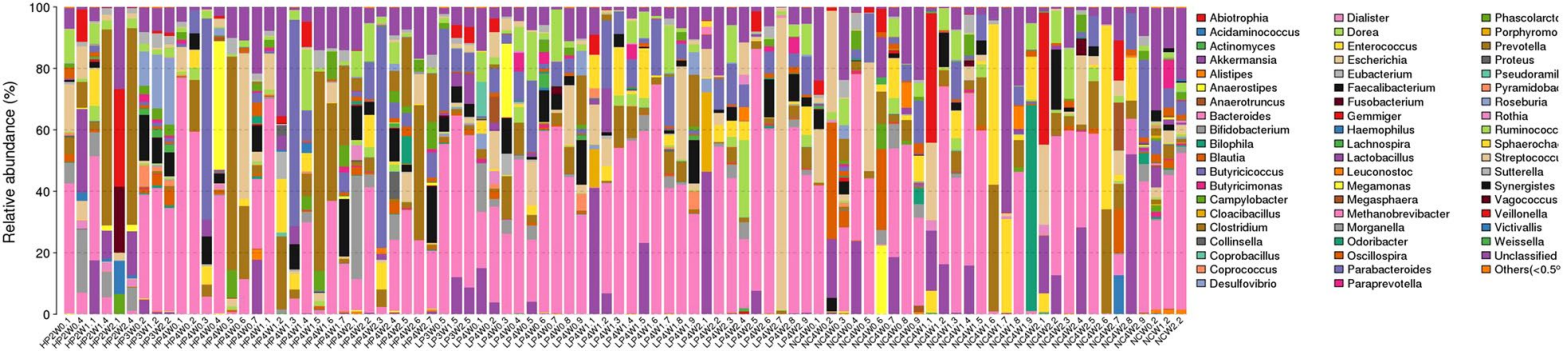
Histogram of the genus profile

In this study, we detected 15 phyla, 17 classes, 19 orders, 38 families, and 56 genera from the 90 faecal samples (Figs. 6, 7, 8, 9, 10). At the phylum level, the faecal samples from the three groups mainly consisted of Bacteroidetes, Firmicutes, Proteobacteria, Actinobacteria, and Verrucomicrobia. Among these phyla, Bacteroidetes and Firmicutes accounted for more than 90% of all bacterial flora at the phylum level. In addition, there were some phyla present at extremely low proportions, such as Acidobacteria, Fusobacteria, Cyanobacteria, Deferribacteres, Euryarchaeota, and Synergistetes. The above results also explain the non-uniform species distribution of the three groups on the OTU rank curve.

**Fig. 11:**
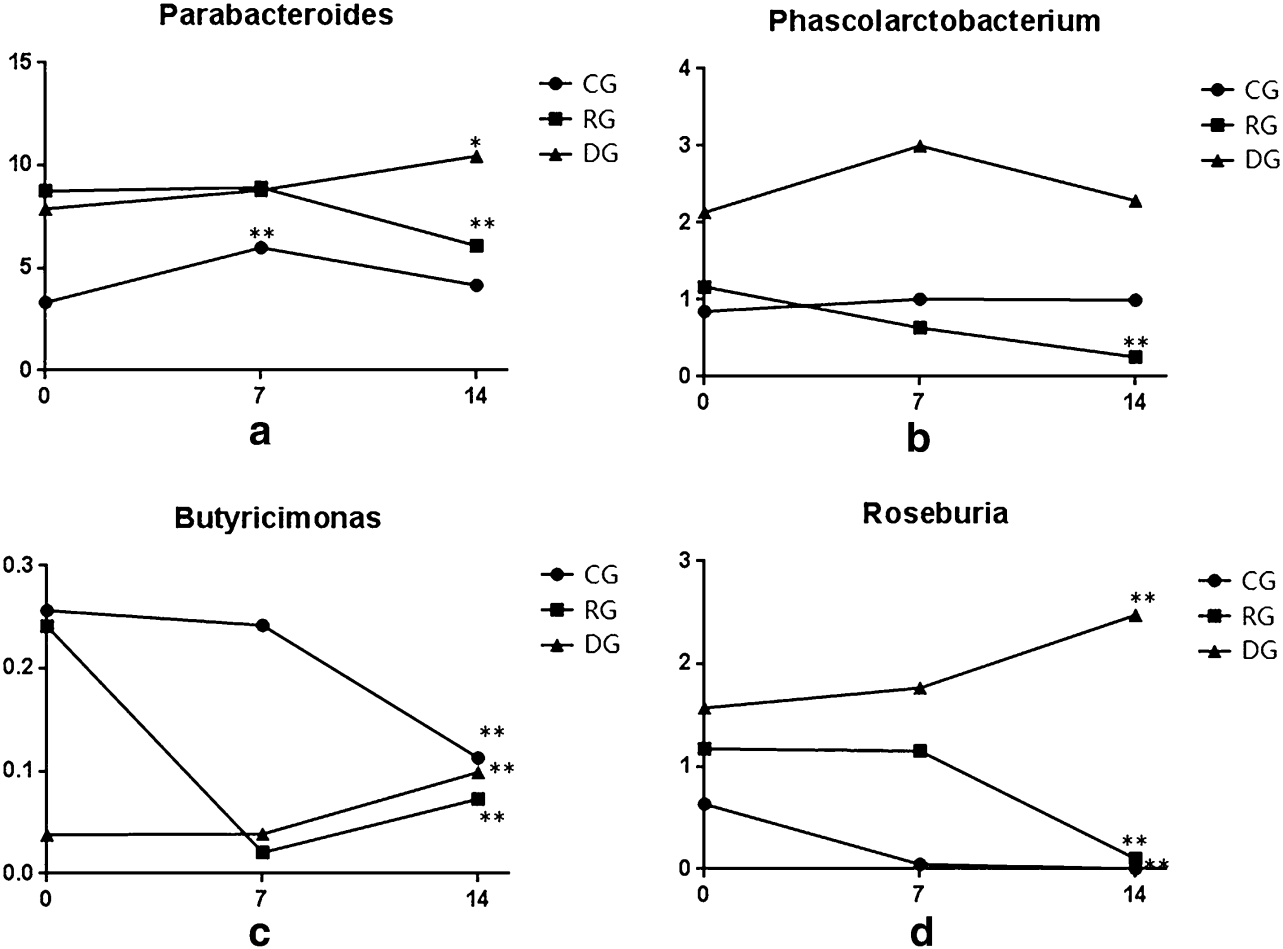
Changes in Parabacteroides, Phascolarctobacterium and butyrate-producing bacteria in the three groups.* CG* control group,* RG* regular group,* DG* double-dosage group. *P < 0.05 and **P < 0.01 compared with the pre-intervention levels

Through the analysis at the phylum, class, order, family, and genus levels, we found that the genus *Parabacteroides* showed an increasing trend from before the intervention to day 7 after the intervention in the control, regular, and double-dosage groups, but there were significant decreases in the regular groups on day 14 compared with the pre-intervention levels (P < 0.01, Fig. 11). However, *Parabacteroides* still showed a significant increase on day 14 in the double-dosage group compared to the pre-intervention levels, and the difference before and after intervention was statistically significant (P < 0.05). The abundance of the genus *Phascolarctobacterium* was increased on day 14 compared with before the intervention in the control group. However, in the regular group, the abundance of this genus was significantly reduced on day 14 compared with before the intervention, and this difference was statistically significant (P < 0.01) (Fig. 11). At the genus level, the abundance of the butyrate-producing bacteria Butyricimonas was significantly reduced on day 14 in the control and regular groups but was significantly increased in the double-dosage group compared to the levels before the intervention (P < 0.01). Similarly, the abundance of some other butyrate-producing bacteria (genus *Roseburia* in the family Lachnospiraceae) in the control and regular groups was decreased on day 14 compared to the levels before the intervention; however the levels were increased in the double-dosage group, which had a higher abundance than before the intervention (P < 0.01, Fig. 11).

## Discussion

The results of this study show that probiotic treatment can reduce the incidence of gut dysbiosis and antibiotic-induced diarrhoea. AIGD can result in diarrhoea, pseudomembranous colitis, and other acute illnesses in a short period of time; in addition, AIGD can also result in obesity, asthma, inflammatory bowel disease, and other chronic diseases that affect health [1, 16, 17]. Antibiotic-associated diarrhoea will disappear when antibiotics are discontinued, and this is intimately associated with the gradual restoration of the gut microbiota balance [1, 9, 11, 12, 17].

Heinsen et al.[18] carried out a study on three healthy adults (20–22-years-old) who took oral antibiotics for a short period of time. They found that most gut bacteria recover to normal levels within 42 days of drug discontinuation. In addition, Perez-Cobas et al.[19] used the 16S rDNA for dynamic monitoring of gut microbiota of one healthy subject who was given intravenous antibiotics. They found significant fluctuations in the gut microbiota: the diversity of the Gram-negative bacteria started to decrease on day 6 and showed a complete decrease on day 11, before gradually recovering on day 40 after the antibiotics were discontinued. Therefore, AIGD shows dynamic changes, and few bacterial species do not easily recover after antibiotics are discontinued.

In this study, the incidence of AIGD in the control group gradually increased to 21.88% within 7 days and to 25% on days 8–14. The gut microbiota OTUs decreased, with a reduction of 24.16% on day 14 compared to that before the use of antibiotics. However, the changes in the structural diversity of the gut microbiota were not significant. Other studies have also found that the use of antibiotics decreases the species abundance of the gut microbiota but does not significantly affect the species diversity in the gut microbiota [18, 19]. The control group didn’t receive any probiotics. The diagnosis of antibiotic-related dysbacteriosis is based on self-report, the symptoms that appear are diarrhea, etc., and there are objective laboratory indicators, so there is no or very unlikely placebo effect.

BIFICO can improve the gut microecological environment, directly participate in the formation of a biological barrier, and resist many pathogenic bacteria [20]. In this study, after BIFICO administration, we found that the incidence of AIGD at all timepoints was lower than that of the control group. In addition, high doses of BIFICO could prevent the occurrence of AIGD more effectively and earlier, and BIFICO could stabilize the species abundance of the gut microbiota. The results reported by Evans et al.[21] are consistent with our findings. The authors demonstrated that the incidence of antibiotic-associated diarrhoea at one week after probiotic administration was significantly lower than that of the placebo group. In contrast, Allen et al.[22] revealed that probiotics (containing *Lactobacillus* and *Bifidobacteria*) did not reduce the incidence of antibiotic-associated diarrhoea on week 8 or of *Clostridium difficile* enteritis on week 12. When antibiotics are used for a short period of time, the most severe destruction to the gut microbiota occurs on day 14, and the gut microbiota usually recovers within 42 days[19]. Therefore, it is more reasonable to examine the results within 14 days of antibiotic usage to determine whether probiotics can prevent AIGD.

We found changes in some bacterial genera in the antibiotic group prior to the probiotic intervention compared with after probiotic intervention. The abundance of *Parabacteroides* gradually decreased in the control group. After high doses of BIFICO were administered, the abundance of *Parabacteroides* remained stable. Wang et al.[23] found that *Parabacteroides* and vitamin D receptor jointly participate in bile metabolism. *Phascolarctobacterium* has a high colonization rate and abundance in the human gut, can produce short-chain fatty acids such as acetic acid and pyruvic acid and is positively correlated with the metabolic status and positive emotions in the host [24, 25]. In this study, we found that the abundance of *Phascolarctobacterium* gradually decreased after the intervention with a regular dosage of BIFICO. After high doses of BIFICO were administered, the abundance of *Parabacteroides* increased. The cause of decreasing abundance of *Parabacteroides* after the intervention with a regular dosage of BIFICO needs further research. Butyrate-producing bacteria are an important functional community amongst the gut microbiota in the human body. The butyrate produced by these bacteria provides a better energy source for the intestinal epithelium, stimulates the generation of regulatory T-cells, inhibits inflammation, and regulates the gene expression of histone deacetylase inhibitors [26, 27]. After antibiotics are used, the levels of butyrate-producing bacteria gradually decline, but the intervention with high doses of BIFICO resulted in a gradual increase of butyrate-producing bacteria. Similarly, Rios et al.[28] conducted co-culture experiments with *Faecalibacterium prausnitzii* and *Bifidobacterium* and found that the latter can promote the former to produce more butyrate.

Our trial has potential limitations. First, this was a single-centre study, and only ten patients were selected from each group to test the gut microbiota by 16S rDNA sequencing. The participants had other conditions and infections in various body sites. These infections, impacting inflammation levels in the host, would differentially affect the gut microbiome before antibiotic and BIFICO intervention. These variable levels of inflammation could contribute to the high variability seen in the results. However, there was no statistical difference among the infection sites and gradients in different groups. Second, although all patients took beta-lactam antibiotics, the antibacterial strength of the various antibiotics is inconsistent. Third, it might be better to show the averages rather than each curve in the figures, but the figures also showed more individual information than the averages and were accepted in many articles [29, 30].

## Conclusion

In conclusion, the probiotic BIFICO might reduce the occurrence of AIGD in a dose-dependent manner. Antibiotics reduced the abundance of the gut microbiota, but the prophylactic use of BIFICO might stabilize the gut microbiota balance.

## Data Availability

The analysed data sets generated during the study are available from the corresponding author on reasonable request.

## Abbreviations

AIGD: Antibiotic-induced gut dysbiosis
DNA: Deoxyribonucleic acid
ANOVA: Analysis of variance
OUT: Operational taxonomic unit
